# Supplementary material for: Correction: A Rapid Lateral Flow Immunoassay for the Detection of Tyrosine Phosphatase-Like Protein IA-2 Autoantibodies in Human Serum
Source: PLoS One. 2014 Aug 25;9(8):e106611. doi: 10.1371/journal.pone.0106611 (PMC4143400; doi:10.1371/journal.pone.0106611)
Supplement: File S1 — Originally published, uncorrected article. (PDF) [file pone.0106611.s001.pdf]

OPEN ACCESS Freely available online

PLOS ONE

A Rapid Lateral Flow Immunoassay for the Detection of Tyrosine Phosphatase-Like Protein IA-2 Autoantibodies in Human Serum

Ingrid Kikkas[1], Roberto Mallone[2,3,4], Etienne Larger[3,4], Hervé Volland[1], Nathalie Morel[1]*

1 CEA, iBiTec-S, Service de Pharmacologie et d'Immunoanalyse, Laboratoire d'Etudes et de Recherches en Immunoanalyse, Gif sur Yvette, France, **2** INSERM, U1016, Cochin Institute, DeAR Lab, Paris, France, **3** Paris Descartes University, Sorbonne Paris Cité, Faculté de Médecine, Paris, France, **4** Assistance Publique Hôpitaux de Paris, Hôpital Cochin, Service de Diabétologie, Paris, France

Abstract

Type 1 diabetes (T1D) results from the destruction of pancreatic insulin-producing beta cells and is strongly associated with the presence of islet autoantibodies. Autoantibodies to tyrosine phosphatase-like protein IA-2 (IA-2As) are considered to be highly predictive markers of T1D. We developed a novel lateral flow immunoassay (LFIA) based on a bridging format for the rapid detection of IA-2As in human serum samples. In this assay, one site of the IA-2As is bound to HA-tagged-IA-2, which is subsequently captured on the anti-HA-Tag antibody-coated test line on the strip. The other site of the IA-2As is bound to biotinylated IA-2, allowing the complex to be visualized using colloidal gold nanoparticle-conjugated streptavidin. For this study, 35 serum samples from T1D patients and 44 control sera from non-diabetic individuals were analyzed with our novel assay and the results were correlated with two IA-2A ELISAs. Among the 35 serum samples from T1D patients, the IA-2A LFIA, the in-house IA-2A ELISA and the commercial IA-2A ELISA identified as positive 21, 29 and 30 IA-2A-positive sera, respectively. The major advantages of the IA-2A LFIA are its rapidity and simplicity.

Citation: Kikkas I, Mallone R, Larger E, Volland H, Morel N (2014) A Rapid Lateral Flow Immunoassay for the Detection of Tyrosine Phosphatase-Like Protein IA-2 Autoantibodies in Human Serum. PLoS ONE 9(7): e103088. doi:10.1371/journal.pone.0103088

Editor: Leonardo A. Sechi, Universita di Sassari, Italy

Received April 15, 2014; **Accepted** June 27, 2014; **Published** July 21, 2014

Copyright: © 2014 Kikkas et al. This is an open-access article distributed under the terms of the Creative Commons Attribution License, which permits unrestricted use, distribution, and reproduction in any medium, provided the original author and source are credited.

Data Availability: The authors confirm that all data underlying the findings are fully available without restriction. All relevant data are within the paper.

Funding: The authors have no support or funding to report.

Competing Interests: The authors have declared that no competing interests exist.

* Email: nathalie.morel@cea.fr

Introduction

Type 1 diabetes (T1D) is an autoimmune disease characterized by the destruction of pancreatic beta cells within the islets of Langerhans. In the course of this autoimmune process, autoantibodies are generated against several beta-cell antigens, e.g. insulin, glutamic acid decarboxylase (GAD65), tyrosine phosphatase-like protein (IA-2) and zinc transporter 8 (ZnT8). At least 1 autoantibody against one of these antigens is present in >95% of individuals with T1D upon hyperglycemia detection [1,2]. These autoantibodies can serve as early markers of T1D, since they can be present years before disease onset [3], allowing for an early diagnosis before clinical manifestations.

Tyrosine phosphatase-like protein IA-2 autoantibodies (IA-2As) are one of the 4 major islet autoantibodies for the diagnosis of T1D. IA-2 is a transmembrane protein and a member of the protein tyrosine phosphatase family. The predominant autoreactive epitopes are in its C-terminal region and IA-2As have been shown to react only with the intracellular part of the protein [4]. IA-2As are detected in approximately 60% of individuals with new-onset T1D [5]. IA-2As are also the autoantibodies with stronger predictive value for impending T1D onset in at-risk individuals, a feature probably linked to their later appearance compared with anti-insulin and anti-GAD autoantibodies [6–8]. One of the current methods for the detection of IA-2As is radioimmunoassay (RIA), which is based on immunoprecipitation of ^{125}I- or ^{35}S-methionine-labeled recombinant IA-2 (intracellular portion) [9,10]. Although several IA-2 RIAs have been reported to achieve high levels of sensitivity and specificity [11], they are expensive and usually take more than 24 h to carry out. In addition, they have the disadvantage of requiring special precautions and licensing because radioactive isotopes are used. Other methods for the detection of IA-2As use enzyme-linked immunosorbent assays (ELISAs) and time-resolved fluorescence assays, in which the immobilized antigen captures autoantibodies from the sample and detection is achieved using labeled antigen [12–14]. Even though these assays do not require radiolabeled compounds, commercially available ELISAs are relatively time-consuming and expensive and still need specialized equipment. There is then a need for assays for detecting autoantibodies to IA-2 that are rapid, easy to use, inexpensive and easily implementable in most clinical laboratories without any special expertise or equipment.

We describe the development of a double antigen bridging lateral flow immunoassay (LFIA) for the detection of *IA-2As in human serum samples*. This immunochromatographic assay uses colloidal gold nanoparticles to visualize the reaction and is performed within 45 min. For the present study, 35 serum samples from patients with newly diagnosed T1D and 44 control sera from non-diabetic individuals were analyzed. To obtain quantitative results, the intensity of each test line was measured using an ESEQuant LFIA Reader from Qiagen. The sensitivity

and specificity of the LFIA were compared with those of our in-house IA-2 bridging ELISA, as well as those of a commercial IA-2A ELISA kit from RSR Ltd.

Materials and Methods

Serum samples

35 serum samples from newly diagnosed T1D patients (11 males, 24 females; mean age 45.5 years; range 18–69) identified positive for IA-2As using RIA (RSR, showed 100% specificity and 70% sensitivity in the 2005 DASP study) and 44 control sera from non-diabetic individuals (28 males, 16 females; mean age 35.7 years; range 24–62) were analyzed. All control samples were non-diabetic with normal blood glucose levels. The study was approved by the local ethics committee CPP Ile de France III. Written consent was obtained from all participants.

Reagents and apparatus

Biotinamidohexanoic acid N-hydroxysuccinimide ester (NHS-LC-biotin), streptavidin and gold chloride solution were from Sigma-Aldrich (Saint Louis, MO, USA). Zeba Spin Desalting Column and the Ultra tetramethylbenzidine (TMB)-ELISA Substrate Solution were from Thermo Fisher Scientific Inc. (Rockford, IL, USA). The streptavidin poly-horseradish peroxidase (HRP) conjugate was from Pierce (Rockford, IL, USA). The nitrocellulose membranes (Prima 40), the sample and absorption pads (standard 14 and Cellulose grade 470, respectively) were from Whatman (Dassel, Germany).

When performing immunoassays, all reagents were diluted in enzyme immunoassay (EIA) buffer, i.e. 0.1 M phosphate buffer pH 7.4 containing 0.15 M NaCl, 0.1% bovine serum albumin (BSA) and 0.01% sodium azide, except for streptavidin poly-HRP conjugate dilution, which was diluted in EIA buffer without addition of sodium azide. Plates were washed with washing buffer (0.01 M phosphate buffer pH 7.4 containing 0.05% Tween 20).

Immunometric assays were performed using an automatic plate washer (Bio Tek ELX405, Winooski, VT, USA) and automatic plate reader (Multiskan Bichromatic). 96-well microtiter plates (Maxisorp) were from Nunc (Roskilde, Denmark).

Expression and purification of IA-2 and IA-2-HA-Tag

A synthetic DNA sequence encoding for the IA-2 intracellular domain (IA-2ic) (amino acids 606–979) and a C-terminal polyhistidine-tag or an HA-Tag were from GeneCust (Dudelange, Luxembourg). Each IA-2ic DNA fragment was inserted in the *E. coli* expression vector pET22b(+) (Novagen, Germany). *E. coli* BL21 (DE3) cells transformed with IA-2ic-pET22b were grown in Luria broth media supplemented with 100 µg/mL ampicillin until the absorbance at 600 nm reached an optical density of 0.6. Subsequently, protein expression was induced with 1 mM isopropyl-β-D-thiogalactopyranoside (IPTG, Sigma) for 4 h. The cells were harvested by centrifugation and resuspended in buffer A (50 mM potassium phosphate buffer, 300 mM NaCl) supplemented with protease inhibitor (Pefabloc, Roche Diagnostics, France). The cells were disrupted by sonication (1 min at 14 W) and the bacterial lysate was centrifuged at 14,000 g for 20 min at 4°C. IA2ic protein was then purified using chelating Sepharose Fast Flow (Pharmacia) charged with nickel as described by the manufacturer.

Biotinylation of IA-2

Biotin was covalently linked to IA-2ic at a 50:1 molar ratio by reaction of an activated N-hydroxysuccinimide ester of biotin with the primary amino groups of the protein. The activated ester was dissolved in dimethylformamide (DMF) and added to a 0.1 M (pH 9.0) borax buffer solution of the protein. After 2-h incubation at room temperature, the reaction was stopped with addition of 1 M Tris-HCl buffer (pH 8.0). Zeba Spin Desalting Columns (Thermo Fisher Scientific) were used to eliminate the unbound biotinylation reagent.

Preparation of the detection conjugate

The colloidal gold nanoparticle solution was prepared by adding 4 mL of gold chloride and 1 mL of 1% sodium citrate solution to 40 mL of boiling water with constant shaking. After the appearance of a purple color, the preparation was allowed to cool to 20°C and stored at 4°C in the dark. Then, 25 µg of streptavidin was added to 1 mL of colloidal gold solution before mixing with 100 µL of 20 mM borax buffer, pH 9.3. The reaction mixture was incubated for 1 h at 20°C, leading to the ionic adsorption of streptavidin on the surface of the colloidal gold particles. Then, 100 µL of 20 mM borax buffer, pH 9.3, containing 1% BSA was added and the mixture was centrifuged at 15,000 *g* for 50 min at 20°C. The supernatant was discarded and the pellet was suspended in 250 µL of 2 mM borax buffer, pH 9.3, containing 1% BSA, sonicated for few seconds, and stored at 4°C in the dark.

Preparation of the LFIA strips

The strip (0.5 cm wide; 6 cm long), includes three components stuck to a backing card (sold with the nitrocellulose membrane) (a) a sample pad (0.5 cm long), (b) a nitrocellulose membrane (2.5 cm long), and (c) an absorption pad (3 cm long). The detection zone consists of a test line and a control line. For this purpose, anti-HA-Tag mAb (mAb 12CA5) and anti-polyhistidine mAb (1 mg/mL in phosphate buffered saline (PBS)) were immobilized on the nitrocellulose membrane as the test and control lines, respectively, both dispensed at 1 µL/cm using an automatic dispenser (BioDot Airjet XYZ 3050, Irvine CA, USA). After drying for 1 h at 40°C in an air oven, the membranes were treated with a blocking solution (10 mM potassium phosphate buffer containing 0.15 M NaCl and 0.5% BSA) for 30 min at 20°C. After three washes for 1 min each in ultra-pure water, the membranes were incubated for 20 min at 20°C in 10 mM potassium buffer containing 0.15 M NaCl, 8% glucose and 0.1% Tween 20. Then, the membranes were dried for 15 min at 40°C before sticking the absorption and sample pads to the top and the bottom of the membranes, respectively. The membranes were finally cut into strips 5 mm wide using an automatic programmable cutter (Guillotine Cutting (CM4000), BioDot, Irvine, CA, USA).

Bridging LFIA

Our IA-2A LFIA was formed as double antigen bridging assay. A series of experiments were carried out varying a range of assay parameters in order to establish the optimal conditions. Different concentrations of HA-Tag-labeled and biotin-labeled IA-2 were tested, ranging from 100 to 1000 ng/mL. In addition, different incubation times of reagents with serum sample (no pre-incubation, 15, 30 or 60 min) and different serum volumes and tracer concentrations were compared. The final assay set up is illustrated in Fig. 1. To allow parallel assaying of a large number of samples, 96-well microtiter plates were used for support. Briefly, 10 µL of serum sample was mixed in the well with 18 µL of a 1:1 mixture of biotinylated and HA-Tag-labeled IA-2 (final concentration 900 ng/mL/each) and 7.5 µL of colloidal gold-conjugated streptavidin in EIA buffer. After a 30-min reaction at room temperature, 64.5 µL of LFIA analysis buffer (0.1 M potassium phosphate buffer pH 7.4 containing 0.1% BSA, 0.15 M NaCl, 0.01% NaN_3, and 0.5% Tween 20) was added to the mixture and

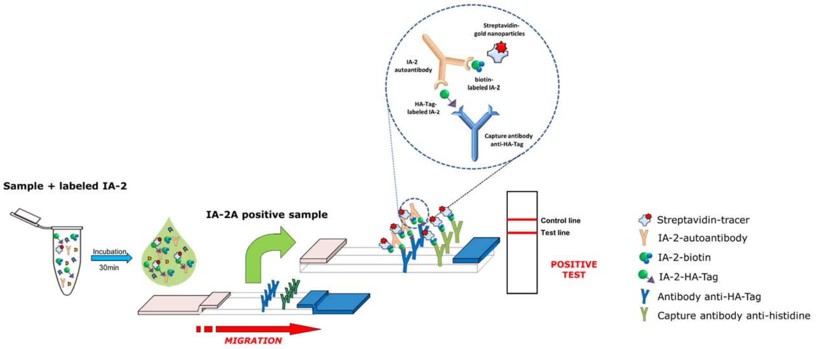

Figure 1. Principle of the IA-2A bridging LFIA. Serum samples are mixed with HA-Tag- and biotin-labeled IA-2 and colloidal gold-conjugated streptavidin before migration along the strip. In the case of a positive test, serum IA-2As form a bridge between HA-Tag- and biotin-labeled IA-2 and this complex is captured by an anti-HA-Tag mAb in the test line. Biotin-labeled IA-2 bound to IA-2As is detected using colloidal gold-labeled streptavidin, producing a purple-colored band on the test line. In the case of a negative test, only the control line is observed. It is generated by the biotinylated IA-2/gold nanoparticle-conjugated streptavidin complex being captured by mAb anti-polyhistidine in the control line. *Insert:* detail of the formation of the bridging complex.
doi:10.1371/journal.pone.0103088.g001

the strip was inserted into the well for the test solution to be migrated by capillary action along the strip. The results were read after 15 min of migration.

Competitive inhibition

Evaluation of the competitive inhibition of IA-2A binding in six IA-2A-positive serum samples was carried out by adding unlabeled IA-2 during the first incubation step with biotinylated and HA-Tag-labeled IA-2. IA-2A signals were consequently detected with the same LFIA format as described above.

Measurement method

The signal intensities of the test and control lines were quantitatively measured using an ESEQuant LFIA Reader (Qiagen, Stockach, Germany). Each strip was placed in a plastic cassette designed to fit the holder of the reader and read individually. The results were analyzed using the Lateral Flow Studio Software (Qiagen).

In-house IA-2A bridging ELISA

All serum samples were further analyzed by an in-house IA-2A bridging ELISA, according to the previously described protocol developed for anti-insulin autoantibodies [15]. Briefly, 96-well microtiter plates were coated with 100 µL/well of mAb anti-HA-Tag (10 µg/mL) in 50 mM phosphate buffer (pH 7.4). After 18 h of incubation at 20°C, plates were washed and blocked with 0.1% BSA-PBS for 24 h at 4°C. Serum samples (25 µL) were mixed with an equal volume of a 1:1 mixture of biotinylated and HA-Tag-labeled IA-2 (final concentration 300 ng/mL/each) in EIA buffer. After incubating for 1 h at room temperature, this solution was transferred into microtiter plates coated with anti-HA-Tag mAb and reacted for 2 h at room temperature on an orbital shaker. The plates were subsequently washed 3 times and 100 µL of 12.5 ng/mL streptavidin poly-HRP solution was added to each well. After 30 min at room temperature followed by 3 washes, 100 µL of Ultra TMB-ELISA Substrate Solution (Pierce) was added to each well. After a 30-min reaction at room temperature, 100 µL of stop solution (0.25 M H_2SO_4) was added to each well. The absorbance was measured at 450 nm.

IA-2 Autoantibody commercial ELISA kit

In addition, all serum samples were analyzed by an IA-2A Version 2 kit (RSR Ltd, Cardiff, UK), using the supplier's protocol. Briefly, 50 µL of serum or calibrator was mixed with 25 µL of reaction enhancer and allowed to interact with IA-2 coated onto ELISA plate wells. After an overnight reaction at 4°C, the well contents were aspirated, the wells washed 3 times with wash buffer (0.15 M NaCl, 20 mM Tris, 0.5 mL/L Tween 20, pH 8.0) and 100 µL of IA-2-biotin was added to each well. After 1-h incubation at room temperature with shaking at 500 shakes/min, the IA-2-biotin solution was aspirated and the wells were washed 3 times. Streptavidin-peroxidase conjugate (100 µL; RSR) was then added and the plates were incubated for 20 min at room temperature with shaking at 500 shakes/min. The wells were then aspirated and washed before addition of TMB (100 µL; RSR). After 20 min in the dark at room temperature, 100 µL of 0.25 M H_2SO_4 was added to stop the reaction and the absorbance of the plate wells was read at 405 nm and 450 nm. Assay calibrators were used to establish the cut-off of the assay. In the DASP 2010 validation, the RSR IA-2A ELISA Version 2 kit showed 100% (n = 90) specificity and 64% (n = 50) sensitivity.

Results

Assay optimization

Since our IA-2A LFIA was designed as a double antigen bridging assay, two tags were required to implement this format (cf. Fig. 1). In the current assay, IA-2As form a bridge between biotinylated IA-2 on one binding site of IA-2A and HA-Tag-labeled IA-2 on the other. The HA-Tag allows this complex to be captured by the anti-HA-Tag mAb coated on the test line of the strip, while the biotin allows the complex to be detected by the colloidal gold nanoparticle-conjugated streptavidin tracer. Under these conditions, if IA-2As are present in the serum, they will react with the biotinylated and HA-Tag-labeled IA-2. The biotin in this complex will in turn bind to the gold nanoparticle-conjugated streptavidin tracer, during the 30-min pre-incubation time in the well. This complex will flow through the absorbent device during the migration process and bind to the capture antibody on the test line, forming a gold nanoparticle-conjugated bridging complex and producing a purple-colored band. As our recombinant IA-2 protein is poly-histidine-tagged, a colored line in the control region

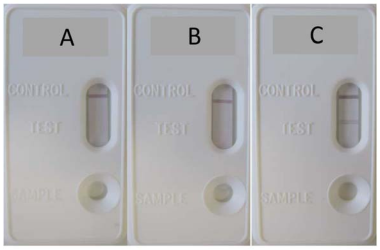

Figure 2. Representative image of negative and positive results with the IA-2A LFIA. A positive signal limited to the control line is obtained with buffer alone (A) and with an IA-2A-negative sample (B). A positive signal in both the test and control line is observed with a IA-2A-positive serum (C).
doi:10.1371/journal.pone.0103088.g002

is produced when biotinylated IA-2 complexed to gold nanoparticle-conjugated streptavidin binds to immobilized anti-polyhistidine mAb. The absence of the colored band in the control region is an indication of an invalid process and the result is not taken into account. Control experiments of the strips with buffer alone or with a control serum produce a purple band in the control area on the device, but no band in the test area. When IA-2A-positive serum samples are used, both test and control lines are detectable on the device, indicating a positive test (Fig. 2).

A bridging immunoassay requires a precise optimization of reagent concentrations. For this reason, different concentrations of HA-Tag-labeled and biotin-labeled IA-2 were tested, ranging from 100 to 1000 ng/mL and the best results were achieved using 900 ng/mL of each reagent. Other optimization steps included the determination of the optimal tracer concentration. Tested concentrations ranged from 1 to 20 µg/mL and the best result was achieved using 7.5 µg/mL (7.5 µL) of colloidal gold nanoparticle-conjugated streptavidin tracer. Finally, different reagent pre-incubation times (no pre-incubation and pre-incubation times ranging from 15 to 60 min) were also investigated and the most suitable results were obtained with 30 min of pre-incubation. The optimized parameters selected for subsequent experiments are described in *Materials and Methods*.

LFIA sensitivity and specificity

In order to validate our IA-2A LFIA, 35 serum samples from patients with newly diagnosed T1D scored as IA-2A-positive by RIA and 44 control sera from non-diabetic individuals were analyzed using the strips. The intensities of the test and control lines were measured using an ESEQuant LFIA Reader. They are associated with the magnitude of the areas of the corresponding peaks. The cut-off value of the IA-2A LFIA was determined via the receiver operator characteristic (ROC) curve resulting from the control samples. Based on the ROC analysis, the area under the curve (AUC) was 0.91 (95% CI 0.83–0.98) and a cut-off value of 1939 intensity units corresponded to 100% specificity and 60% sensitivity. According to this cut-off value, IA-2As were detected in 21 out of 35 samples from TD1 patients and in 0 out of 44 healthy controls (Fig. 3).

To confirm the specificity of the assay, competition experiments were performed. Six IA-2A-positive serum samples were incubated with 10 µg/mL of unlabeled IA-2 together with biotinylated and HA-Tag-labeled IA-2 (900 ng/mL/each) during the pre-incubation step. Addition of 10 µg/mL of unlabeled IA-2 completely inhibited IA-2A detection in the positive sera, indicating that the binding of IA-2As to the capture mAb-coated strip surface is specific (data not shown).

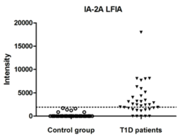

Figure 3. Titer distribution of the serum samples analyzed using the IA-2A LFIA. Sera were from 35 T1D patients and 44 age-matched controls. The dotted line indicates the cut-off value (1939 a.u.) based on the control samples.
doi:10.1371/journal.pone.0103088.g003

Comparison of IA-2A LFIA with in-house IA-2A ELISA and IA-2A ELISA kit from RSR

In order to characterize our in-house IA-2A LFIA further, we compared it with two other non-isotopic assays: the in-house IA-2A bridging ELISA and the IA-2A ELISA kit from RSR (see Methods). The cut-off value of the in-house ELISA was determined as the mean plus 3 SD of the control samples and corresponded to 86 milli-absorbance units (mAU). In addition, the ROC curve was drawn, resulting in an AUC of 0.88 (95% CI 0.80–0.97), and a cut-off value of 90 mAU corresponded to 100% specificity and 82% sensitivity. The cut-off value of the IA-2A ELISA commercial kit was determined according to the manufacturer's recommendations by using assay calibrators. With the in-house ELISA and the RSR ELISA, respectively 29 out of 35 (82%) samples from T1D patients and 30 out of 35 (85%) were identified as positive. Both assays were correlated using regression analysis ($R^2 = 0.7135$; $P < 0.001$; Fig. 4). Among the discrepant results, two samples weakly positive with the commercial ELISA were negative with the in-house IA-2A ELISA, while one sample weakly positive with the in-house ELISA was negative with the commercial ELISA.

Finally, our IA-2A LFIA was compared with the in-house IA-2A bridging ELISA and the results obtained with both assays were correlated using regression analysis ($R^2 = 0.5196$; $P < 0.001$; Fig. 5). 11 samples that were weakly positive with the in-house IA-2A ELISA and also positive for GAD autoantibodies (RIA; RSR; data not shown) were LFIA-negative. Three samples that were weakly positive with the LFIA were negative by in-house ELISA and 2 of these subjects were also GAD autoantibody-positive.

Discussion

Establishing simple and rapid assays for the detection of T1D-associated autoantibodies is useful for screening of individuals at risk for T1D and for diagnostic classification of new-onset diabetes cases. A number of assays are available for the detection of IA-2As in clinical samples, including RIAs and ELISAs. Currently, radioactive liquid-phase assays are the reference method for detection of IA-2As. However, the use of RIA in routine laboratories is limited by the synthesis and handling of the radioactive antigen. Other disadvantages are that both commercially available ELISAs and RIAs are relatively time-consuming (~24 h), expensive and require specialized equipment. Therefore, alternative assay methods for detecting IA-2As are desirable.

Several studies have been published so far measuring antibodies in serum samples using LFIAs [16–18]. To our knowledge, only one article has reported the use of LFIA to assay autoantibodies in serum samples. In this study, autoantibodies against mutated and citrullinated vimentin (MCV), considered as diagnostic marker for rheumatoid arthritis, are measured in serum samples and in whole blood [19]. This assay is based on a conventional direct antibody sandwich format, where anti-MCV autoantibodies bound to

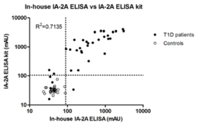

Figure 4. Comparison of IA-2A results detected by in-house IA-2A bridging ELISA and a commercial IA-2A ELISA. Serum samples from 35 T1D patients and 44 control subjects were analyzed with both methods. Dotted lines indicate the cut-off values for both assay methods. mAU, milli-absorbance units.
doi:10.1371/journal.pone.0103088.g004

immobilized antigens at the test line are detected by anti-human IgG gold conjugate.

In the current study, we describe the development of a novel LFIA for detection of IA-2As. To our knowledge, this is the first LFIA format described, detecting T1D-associated autoantibodies in human serum samples. The double antigen bridging LFIA with a pre-incubation step in solution allows to optimize the formation of antigen-autoantibody complex and to increase the specificity of the assay by requiring two specific binding events with IA2 antigen to induce a signal. In the present study, results obtained by IA-2A LFIA were compared with results from IA-2A ELISAs. Both in-house and RSR ELISA were relatively well correlated ($R^2 = 0.7135$; $P<0.001$) and displayed similar sensitivities. Out of 11 samples that were in-house IA-2A ELISA-positive, but IA-2A LFIA-negative, most resulted in weak signals with in-house ELISA. Thus the lower diagnostic sensitivity of LFIA very likely reflects a lower analytical sensitivity which could be possibly explained by the fact that the capture time of the autoantibody-antigen complex is much shorter in the case of the LFIA-based assay (few seconds over the test line) than in the case of ELISA (2-h incubation on the microtiter plates). Although IA-2A LFIA is less sensitive than IA-2A ELISA, it has several advantages. First, IA-2A LFIA is very rapid (45 min) compared with commercial ELISAs and RIAs, and can detect IA-2As from a single drop of serum. This non-radioactive immunochromatographic method does not require any washing steps. Test results can be evaluated visually, and in this case no special equipment is needed, or also quantified using an LFIA reader in order to obtain quantitative results. Taking all of this into account, LFIA can be very useful in clinical practice in yielding an initial rapid IA-2A assessment or for routine screening of individuals at risk of developing T1D, since T1D-associated autoantibodies can be present several months or even years before disease onset. Future experiments are envisaged to develop similar LFIA formats using whole blood rather than serum. This could further facilitate the screening and diagnosis of patients, as the test could be performed with a drop of blood directly in the medical office.

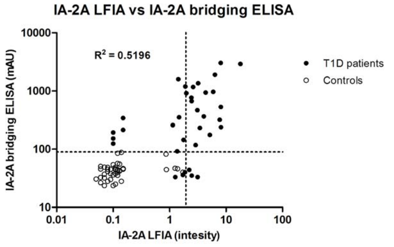

Figure 5. Comparison of IA-2A levels obtained by IA-2A LFIA and in-house IA-2A bridging ELISA. Serum samples from 35 T1D patients and 44 control subjects were analyzed with both methods and the two assays were correlated. Dotted lines indicate the cut-off values for both assay methods. mAU, milli-absorbance units.
doi:10.1371/journal.pone.0103088.g005

Several longitudinal studies following T1D patients and their relatives have shown that the presence of multiple autoantibodies (GAD65, insulin and ZnT8 in addition to IA-2) has the highest positive predictive value for T1D [20,21]. Although not everyone with autoantibodies progresses to T1D, the risk increases with the number of antibody types, with three to four antibody types giving a risk of progressing to T1D of 60%–100% [22]. This is why developing an assay, where multiple diabetes-associated autoantibodies would be measured simultaneously, is of very high interest. We propose that the same LFIA approach, as described in this article, could be adapted to the simultaneous detection of other islet autoantibodies on the same test strip. For this capture antibodies with very high affinity are needed.

In conclusion, we have developed a novel LFIA based on a bridging format which enables rapid and practical detection of IA-2As in human serum samples. Its key advantages are its simplicity and its fast readout (45 min), which means that our IA-2A LFIA could easily be used for preliminary screening of at-risk individuals and for diagnostic assessment of T1D directly in an outpatient hospital setting.

Acknowledgments

We would like to thank Dr. Christophe Creminon, Dr. Catherine Vuillemard, Dr. Xavier Millot and Sophie Volturo for their help. Ingrid Kikkas obtained a grant from the PhD program of the Commissariat à l'Energie Atomique.

Author Contributions

Conceived and designed the experiments: IK NM. Performed the experiments: IK NM. Analyzed the data: IK NM HV RM. Contributed reagents/materials/analysis tools: IK NM HV RM EL. Contributed to the writing of the manuscript: IK NM RM.

References

1. Ziegler AG, Rewers M, Simell O, Simell T, Lempainen J, et al. (2013) Seroconversion to multiple islet autoantibodies and risk of progression to diabetes in children. JAMA 309: 2473–2479.
2. Sacks DB, Arnold M, Bakris GL, Bruns DE, Horvath AR, et al. (2011) Guidelines and recommendations for laboratory analysis in the diagnosis and management of diabetes mellitus. Clin Chem 57: e1–e47.
3. Jahromi MM, Eisenbarth GS (2007) Cellular and molecular pathogenesis of type 1A diabetes. Cell Mol Life Sci 64: 865–872.
4. Lampasona V, Bearzatto M, Genovese S, Bosi E, Ferrari M, et al. (1996) Autoantibodies in insulin-dependent diabetes recognize distinct cytoplasmic domains of the protein tyrosine phosphatase-like IA-2 autoantigen. J Immunol 157: 2707–2711.
5. Winter WE, Schatz DA (2011) Autoimmune markers in diabetes. Clin Chem 57: 168–175.
6. Decochez K, De Leeuw IH, Keymeulen B, Mathieu C, Rottiers R, et al. (2002) IA-2 autoantibodies predict impending type I diabetes in siblings of patients. Diabetologia 45: 1658–1666.
7. Achenbach P, Warncke K, Reiter J, Naserke HE, Williams AJ, et al. (2004) Stratification of type 1 diabetes risk on the basis of islet autoantibody characteristics. Diabetes 53: 384–392.
8. Achenbach P, Hummel M, Thümer L, Boerschmann H, Höfelmann D, et al. (2013) Characteristics of rapid vs slow progression to type 1 diabetes in multiple islet autoantibody-positive children. Diabetologia 56: 1615–1622.
9. Morgenthaler NG, Löbner K, Morgenthaler UY, Christie MR, Seissler J, et al. (1998) Recombinant IA-2 expressed in E. coli can be used for the routine detection of autoantibodies in Type-I diabetes. Horm Metab Res 30: 559–564.
10. Masuda M, Powell M, Chen S, Beer C, Fichna P, et al. (2000) Autoantibodies to IA-2 in insulin-dependent diabetes mellitus. Measurements with a new immunoprecipitation assay. Clin Chim Acta 291: 53–66.

11. Törn C, Mueller PW, Schlosser M, Bonifacio E, Bingley PJ, et al. (2008) Diabetes Antibody Standardization Program: evaluation of assays for autoantibodies to glutamic acid decarboxylase and islet antigen-2. Diabetologia 51: 846–852.
12. Westerlund-Karlsson A, Suonpää K, Ankelo M, Ilonen J, Knip M, et al. (2003) Detection of autoantibodies to protein tyrosine phosphatase-like protein IA-2 with a novel time-resolved fluorimetric assay. Clin Chem 49: 916–923.
13. Chen S, Willis J, Maclean C, Ananieva-Jordanova R, Amoroso MA, et al. (2005) Sensitive non-isotopic assays for autoantibodies to IA-2 and to a combination of both IA-2 and GAD65. Clin Chim Acta 357: 74–83.
14. Löbner K, Khoo-Morgenthaler UY, Seissler J, Morgenthaler NG, Scherbaum WA (1999) Detection of autoantibodies to the diabetes-associated antigen IA-2 by a sensitive enzyme-linked immunosorbent assay. Horm Metab Res 31: 686–691.
15. Kikkas I, Mallone R, Tubiana-Rufi N, Chevenne D, Carel JC, et al. (2013) A simple and fast non-radioactive bridging immunoassay for insulin autoantibodies. PLoS One 8: e69021.
16. Biagini RE, Sammons DL, Smith JP, MacKenzie BA, Striley CA, et al. (2006) Rapid, sensitive, and specific lateral-flow immunochromatographic device to measure anti-anthrax protective antigen immunoglobulin g in serum and whole blood. Clin Vaccine Immunol 13: 541–546.
17. Mdluli P, Tetyana P, Sosibo N, van der Walt H, Mlambo M, et al. (2014) Gold nanoparticle based Tuberculosis immunochromatographic assay: The quantitative ESE Quanti analysis of the intensity of test and control lines. Biosens Bioelectron 54: 1–6.
18. Silpasakorn S, Srisamut N, Ekpo P, Zhang Z, Chao CC, et al. (2012) Development of new, broadly reactive, rapid IgG and IgM lateral flow assays for diagnosis of scrub typhus. Am J Trop Med Hyg 87: 148–152.
19. Renger F, Bang H, Feist E, Fredenhagen G, Natusch A, et al. (2010) Immediate determination of ACPA and rheumatoid factor–a novel point of care test for detection of anti-MCV antibodies and rheumatoid factor using a lateral-flow immunoassay. Arthritis Res Ther 12: R120.
20. Parikka V, Näntö-Salonen K, Saarinen M, Simell T, Ilonen J, et al. (2012) Early seroconversion and rapidly increasing autoantibody concentrations predict prepubertal manifestation of type 1 diabetes in children at genetic risk. Diabetologia 55: 1926–1936.
21. Elding Larsson H, Vehik K, Gesualdo P, Akolkar B, Hagopian W, et al. (2014) Children followed in the TEDDY study are diagnosed with type 1 diabetes at an early stage of disease. Pediatr Diabetes 15: 118–126.
22. Knip M (2003) Environmental triggers and determinants of beta-cell autoimmunity and type 1 diabetes. Rev Endocr Metab Disord 4: 213–223.
